# Successful Laparoscopic Resection of a Giant Para-aortic Pheochromocytoma: A Case Report

**DOI:** 10.7759/cureus.90215

**Published:** 2025-08-16

**Authors:** Loay Ghalyoun, Diya E Viju, Khadiga Abdelmonem, Fahed S Khdrawe, Joaquin Picazo-Yeste

**Affiliations:** 1 Medicine, Burjeel Medical City, Abu Dhabi, ARE; 2 Radiology, Burjeel Medical City, Abu Dhabi, ARE; 3 General Surgery, Burjeel Medical City, Abu Dhabi, ARE; 4 Medicine, Elrazi University, Khartoum, SDN

**Keywords:** asymptomatic, giant tumor, laparoscopy, non-functional, paraganglioma, pheochromocytoma

## Abstract

Paragangliomas are rare neuroendocrine tumors that may be functional or non-functional and are typically located in the adrenal or extra-adrenal regions. Surgical resection remains the cornerstone of management, with open surgery generally recommended for large tumors due to anatomical complexity. We report the case of a 51-year-old South Asian man with a non-functional retroperitoneal paraganglioma measuring 9 × 6 × 5.4 cm, located para-aortically from L2 to L4. Despite its size and proximity to critical vascular structures, the tumor was successfully excised laparoscopically. Preoperative imaging included computed tomography (CT), gallium-68 DOTATATE positron emission tomography-CT (PET-CT), and iodine-131 metaiodobenzylguanidine* *(I-131 MIBG) scintigraphy, all supporting the diagnosis. Histopathology confirmed paraganglioma with vascular invasion and a Grading of Adrenal Pheochromocytoma and Paraganglioma (GAPP) score of 6/10. The patient had no classical symptoms of catecholamine excess and recovered well postoperatively. This case demonstrates that, with careful planning and experienced surgical expertise, even large para-aortic paragangliomas can be safely managed laparoscopically, challenging traditional preferences for open surgery in such cases.

## Introduction

Pheochromocytomas are uncommon neuroendocrine tumors that may or may not produce catecholamines. Paragangliomas, which are extra-adrenal neuroendocrine tumors arising from similar chromaffin cells, are distinguished from pheochromocytomas by their location outside the adrenal glands. Common symptoms of such tumors include headache, palpitations, anxiety, and diaphoresis. Since the condition is not specific to any age or gender and can sometimes be asymptomatic, accurate diagnosis using biochemical tests and imaging is essential. Effective management requires a multidisciplinary approach, combining surgery, pharmacotherapy, and, in advanced cases, systemic treatment options (tyrosine kinase inhibitors (TKIs), mammalian target of rapamycin complex 1 (mTORC1) inhibitor everolimus, immunotherapy, cold somatostatin analogs (biotherapy), and radioligand therapy) which are used to optimize outcomes and significantly improve patient prognosis [1].

Pheochromocytomas and paragangliomas (PPGLs) usually require urgent care due to their association with morbidity and mortality. Pheochromocytomas are rare, with an estimated annual incidence of 0.05%, primarily in adults aged 30-50 [2]. The incidence has been increasing over time, largely due to incidental findings on imaging studies [3]. A pheochromocytoma is considered massive when it measures at least 7 cm in diameter [4]. In this article, we present the case of a 51-year-old South Asian man diagnosed with a pheochromocytoma measuring 9 × 6 × 5.4 cm, which was successfully excised laparoscopically, despite the rarity of the tumor and the technical challenges involved.

## Case presentation

A 51-year-old South Asian man presented with a four-day history of constant, dull anterior abdominal pain that worsened with food intake. Clinical examination revealed a deeply palpable, soft mass on the left side of the umbilicus. An initial abdominal ultrasound suggested a retroperitoneal mass, which was confirmed on contrast-enhanced computed tomography (CECT) imaging. The scan demonstrated a sizable, well-defined ovoid heterogeneously enhancing soft tissue density lesion measuring 9 × 6 × 5.4 cm (craniocaudal × transverse × anteroposterior) within the left lower abdomen. The lesion exhibited central non-enhancing areas suggestive of cystic or necrotic degeneration. He was referred to general surgery for further evaluation, and routine laboratory tests, including tumor markers such as chromogranin A, serum metanephrines, and normetanephrines, were ordered. Due to a background of functional dyspepsia, a gastroenterology referral was made for upper endoscopy. Interventional radiology was consulted, and a CT-guided biopsy was planned.

The patient denied classical symptoms of catecholamine excess such as palpitations, anxiety, or sustained hypertension. However, he has dyslipidemia and hyperglycemia. This was supported by normal biochemical lab results (Table 1). Clinical examination revealed a slim build with normal cardiovascular and respiratory findings. Cardiac evaluation, including electrocardiogram (ECG), echocardiography, and stress testing, revealed only early repolarization changes; otherwise, findings were within normal limits.

**Table 1:** Biochemical abnormalities. HbA1c: glycated hemoglobin; VMA: vanillylmandelic acid; TSH: thyroid-stimulating hormone

Test	Values	Reference range
Blood pressure	123/88 mmHg	<140/90 mmHg (optimal)
HbA1c	6%	4-5.6% (normal); 5.7-6.4% (pre-diabetes)
Cholesterol (total)	7.09 mmol/L	<5.2 mmol/L
Triglycerides	4.0 mmol/L	<1.7 mmol/L
Catecholamines-noradrenaline (plasma)	320.4 pg/mL	<600 pg/mL
Catecholamines-adrenaline (plasma)	<8 pg/mL	<125 pg/mL
Metanephrine (urine, 24 hr)	98 µg/24 hr	58-276 µg/24 hr
Normetanephrine (urine, 24 hr)	159 µg/24 hr	156-729 µg/24 hr
VMA (urine, 24 hr)	3.8 mg/24 hr	0.0-7.5 mg/24 hr
TSH	1.25 mIU/L	0.4-4.0 mIU/L

**Figure 1:**
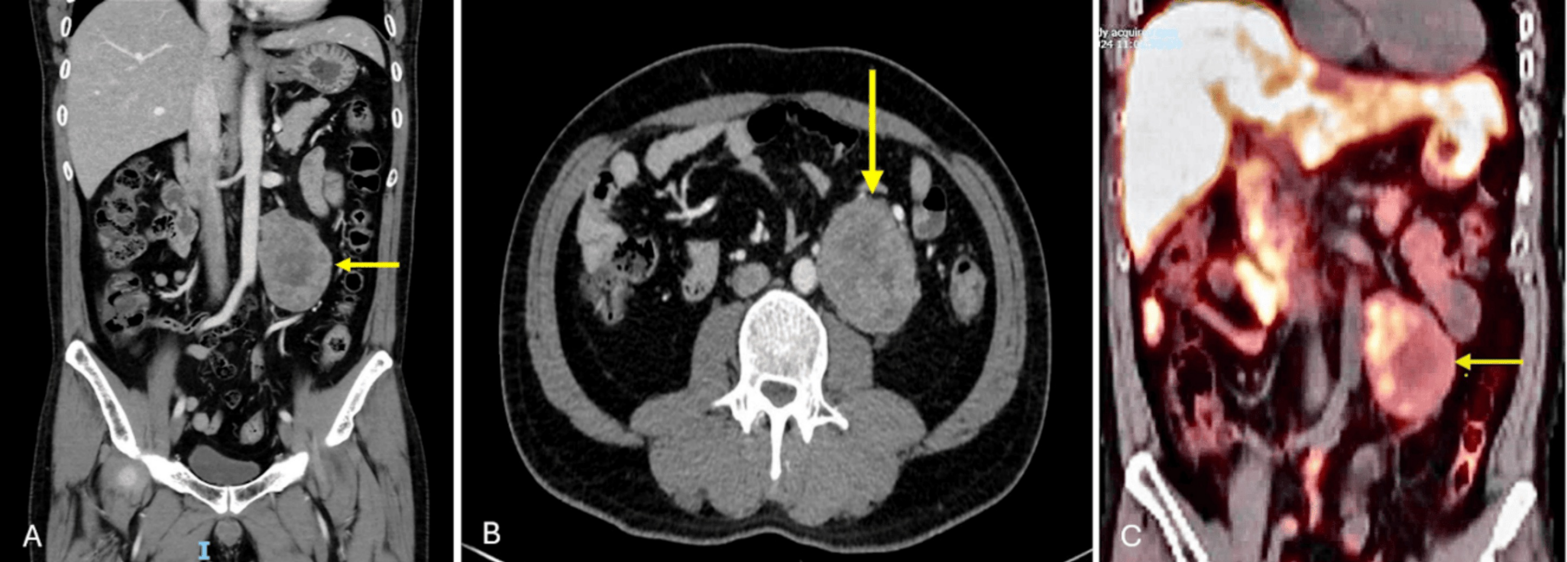
(A and B) Well-defined ovoid soft tissue mass in the left lower retroperitoneum, measuring 9 × 6 × 5.4 cm, with heterogeneous enhancement and central non-enhancing areas consistent with cystic or necrotic changes. (C) A hypermetabolic necrotic mass extending from L2 to L4, closely related to the abdominal aorta and left psoas muscle.

During the gastroenterology evaluation, the patient presented with anemia and constipation. A CT-guided fine-needle core biopsy confirmed a retroperitoneal extra-adrenal paraganglioma (pheochromocytoma) with a Ki-67 proliferation index of <1%. A gallium-68 DOTATATE positron emission tomography-CT (PET-CT) scan revealed a hypermetabolic necrotic mass, extending from the L2 to L4 vertebral levels, abutting the abdominal aorta and resting on the left psoas muscle with a preserved plane of separation (Figure 1). No DOTATATE-avid distant or nodal metastases were detected. Additional incidental findings included a small hemangioma in the D6 vertebral body and focal uptake in the right thyroid lobe; both were deemed clinically insignificant, with the vertebral hemangioma requiring no intervention and the thyroid uptake found to be non-specific on follow-up ultrasound.

Iodine-131 metaiodobenzylguanidine (I-131 MIBG) scintigraphy demonstrated avid tracer uptake in the same para-aortic region, consistent with the known paraganglioma. No additional MIBG-avid lesions were seen. The patient was appropriately prepared with thyroid blockade and received low-dose alpha-blockade therapy before surgery, as advised by endocrinology.

He underwent laparoscopic excision of the para-aortic pheochromocytoma, along with cystoscopy and left ureteral stenting. Under general anesthesia, the patient was positioned supine with a 30-degree left tilt. Pneumoperitoneum was safely established via Palmer's point, and four trocars were placed along a right-sided convex line. A diagnostic laparoscopy confirmed a large retroperitoneal mass arising lateral to the ligament of Treitz and displacing the inferior mesenteric vein, which was identified and ligated using Hem-o-lok clips. Dissection proceeded medially to expose the mass along the aorta and posteriorly to the psoas muscle, with the careful preservation of the left ureter throughout.

**Figure 2:**
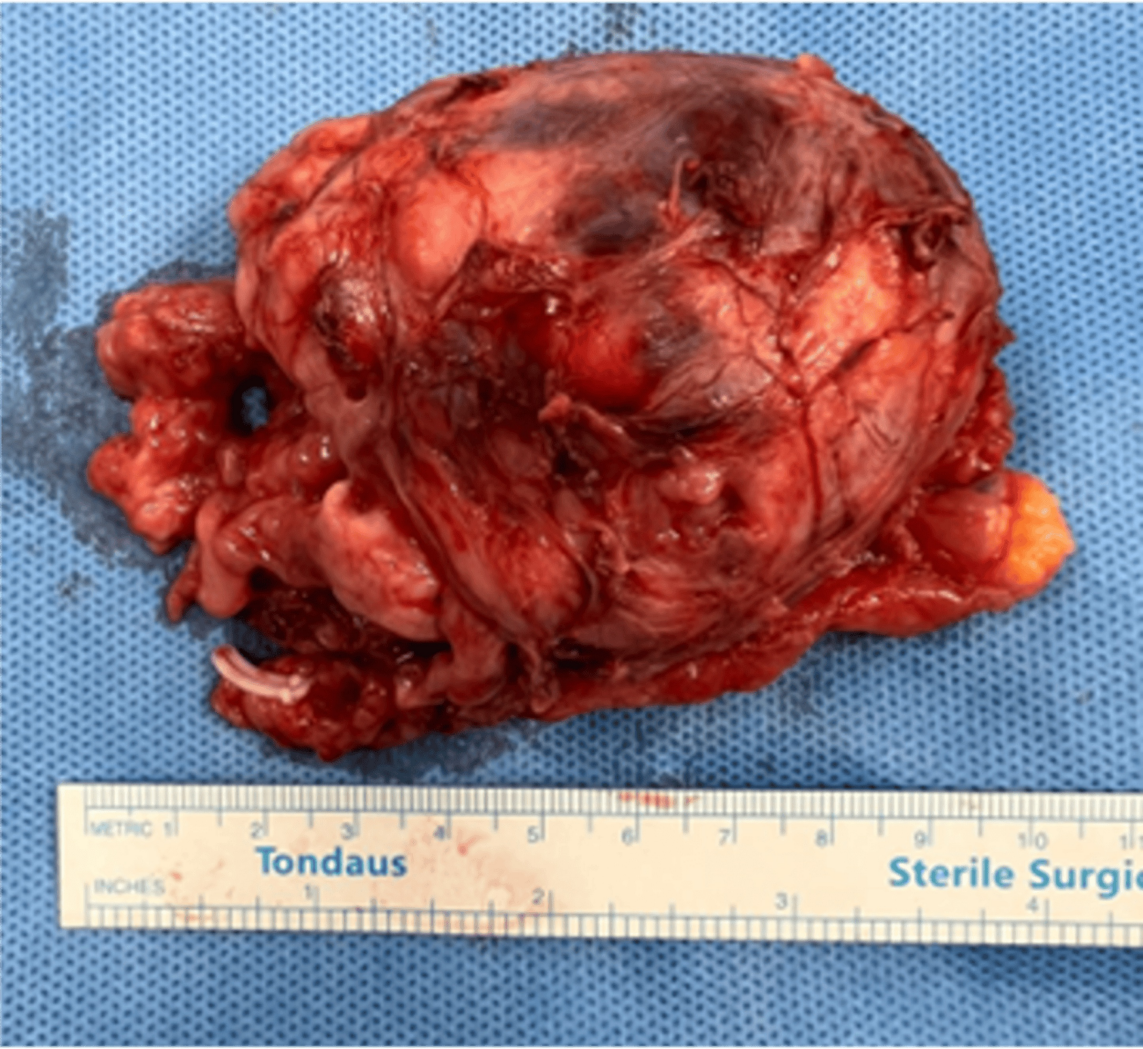
The excised mass was a solid tumor measuring 9 cm in greatest dimension. The lesion exhibited areas of hemorrhage and necrosis, consistent with typical features of a pheochromocytoma.

The mass was mobilized with a harmonic scalpel and a monopolar hook, and radical excision was achieved without capsular rupture (Figure 2). Intraoperatively, the patient experienced a mild rise in systolic blood pressure (not exceeding 150 mmHg), followed by transient hypotension post-excision, which was managed conservatively. Hemostasis was secured with electrocautery and adjunctive use of hemostatic agents. The specimen was retrieved via a Pfannenstiel incision. Blood loss was minimal, and no intraoperative or immediate postoperative complications occurred. The patient was extubated and transferred to the ICU for standard post-pheochromocytoma monitoring.

**Figure 3:**
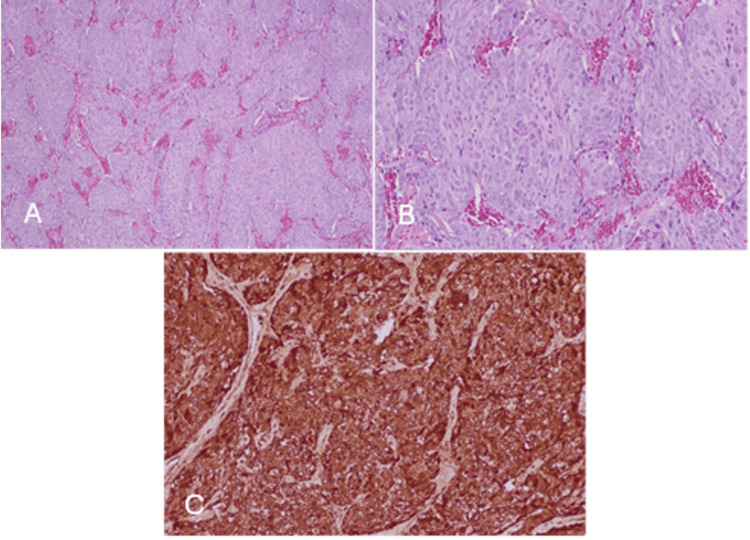
(A) Neoplasm with Zellballen arrangement of neoplastic cells, separated by thin-walled blood vessels (4×, hematoxylin and eosin stain). (B) Neoplastic cells with moderate amount of eosinophilic to amphophilic cytoplasm arranged in nests, separated by thin-walled blood vessels (10×, hematoxylin and eosin stain). (C) Neoplastic cells display diffuse strong staining for chromogranin (10×, immunohistochemistry).

Histopathology confirmed an 8 cm paraganglioma with intracapsular vascular invasion and microscopic involvement of retroperitoneal margins in approximately 10-15% of the circumference. The tumor exhibited classical Zellballen architecture without necrosis or significant mitotic activity. The Grading of Adrenal Pheochromocytoma and Paraganglioma (GAPP) score was 6/10, indicating moderate differentiation and intermediate metastatic risk, which warrants long-term follow-up for at least 10 years. The scoring system combines histological, immunohistochemical, and biochemical parameters to stratify metastatic potential, with scores of 3-6 denoting intermediate risk. Immunohistochemistry showed tumor cell positivity for chromogranin A, synaptophysin, CD56, vimentin, GATA3, and S100, consistent with neuroendocrine origin, which supports the neuroendocrine diagnosis. Upper and lower endoscopies showed no abnormalities (Figure 3).

At the postoperative endocrine follow-up, the patient was normotensive, with catecholamines and metanephrines within normal limits, and he was clinically stable. He was found to be euthyroid and euvolemic, with glycated hemoglobin (HbA1c) of 6.1%, for which dietary counseling was provided. A follow-up PET-CT showed no residual or recurrent disease. He was referred to medical oncology for further management and long-term surveillance.

## Discussion

PPGLs are rare neuroendocrine tumors that develop from chromaffin cells either within the adrenal medulla or from neural crest-derived cells situated outside the adrenal glands [5]. Approximately 85-90% of PPGLs are located in the adrenal gland, while only 10-15% are located in the extra-adrenal and are called paragangliomas [6]. Paragangliomas are rare, with an estimated incidence of approximately one in every 300,000 individuals [7]. 

Most paragangliomas secrete catecholamines, therefore causing the known clinical symptoms, including high blood pressure, tachycardia, headaches, and sweating [8]. Non-functional abdominal tumors are often large at the time of diagnosis, typically presenting with abdominal or back pain and a detectable mass [6]. However, in our case, the tumor was non-functional, and the patient did not exhibit classic symptoms, instead presenting with abdominal pain due to local mass effect, highlighting the variability in clinical presentation. Only 10% of paragangliomas are non-functional; therefore, they are found incidentally through imaging, rather than through clinical presentation [9]. However, several studies report higher variability in the proportion of non-functional tumors depending on tumor location. Non-functional paragangliomas are usually found in the neck and rarely in the abdomen, representing ~10% [6]. Paragangliomas are most commonly diagnosed in individuals between the ages of 20 and 40, with no notable difference in incidence between males and females [9]. Our patient, a 51-year-old man, falls slightly outside the typical age range of 20-40 years for paragangliomas, but his gender aligns with the condition's equal sex distribution. The majority of extra-adrenal paragangliomas are sporadic, with familial cases accounting for about 10%, as seen in our patient [9]. 

In the evaluation of retroperitoneal paragangliomas, imaging plays a pivotal role in diagnosis and surgical planning. In our case, abdominal ultrasonography (USG) was the initial modality that suggested the presence of a retroperitoneal mass. While USG can detect both functional and non-functional lesions, Doppler studies may further characterize the tumor's vascularity [9]. However, CECT of the abdomen remains the preferred diagnostic tool, as it allows for the comprehensive visualization of the sympathetic chain and the identification of small or multiple lesions [6]. In our patient, CT demonstrated a large, heterogeneously enhancing mass with necrotic areas. Functional imaging was further pursued using gallium-68 DOTATATE PET-CT, which confirmed the lesion's neuroendocrine nature and ruled out distant metastases. Notably, gallium-68 DOTATATE PET-CT is known to be more sensitive than I-131 MIBG scintigraphy, especially in detecting non-functional tumors. Additionally, I-131 MIBG scintigraphy showed strong avidity in the lesion, corroborating the diagnosis and aiding in preoperative planning. Although magnetic resonance imaging (MRI) offers superior tissue characterization without radiation, it did not provide a significant additional benefit in our case [9]. Histopathological evaluation confirmed an 8 cm paraganglioma with vascular invasion and a GAPP score of 6/10, indicating intermediate metastatic potential. The GAPP system classifies PPGLs into well-differentiated (0-2), moderately differentiated (3-6), and poorly differentiated (7-10) categories based on histopathological and biochemical criteria. While useful for risk stratification, the GAPP model has limitations and is not fully predictive of patient mortality, despite their high sensitivity for predicting metastasis [10].

Surgical excision is the primary treatment for paragangliomas, while radiotherapy is considered for recurrent cases. Chemotherapy is generally reserved for those with metastatic disease [9]. Here, surgery was the cornerstone of treatment, while radiotherapy and chemotherapy were reserved for recurrent or metastatic disease. Although the open transperitoneal approach is traditionally preferred, laparoscopic resection, while technically challenging, has been reported in limited studies as a safe and feasible alternative, despite longer operative times and the absence of standardized guidelines [11]. In our case, the patient underwent laparoscopic excision of the 9 × 6 × 5.4 cm retroperitoneal paraganglioma, which was located para-aortically from L2 to L4 and abutting the left psoas muscle. Preoperative management included thyroid blockade and low-dose alpha-blockade following current clinical guidelines, despite the tumor being clinically and biochemically non-functional [6]. During surgery, hemodynamic instability, usually hypertensive spikes during tumor manipulation, and hypotension after vessel ligation are well-recognized complications in paraganglioma cases [12]. Our patient experienced only minor intraoperative hypertension and transient hypotension post-resection, both managed conservatively. Postoperatively, the patient remained normotensive with normalized catecholamine levels and was referred for long-term oncologic surveillance.

## Conclusions

This case highlights the diagnostic and therapeutic challenges associated with retroperitoneal paragangliomas, particularly when non-functional and presenting with non-specific symptoms such as abdominal pain. Despite the absence of classical catecholamine-related signs, thorough imaging and biochemical workup enabled the timely diagnosis. Histopathology confirmed the diagnosis of paraganglioma. The use of multimodal imaging, including CECT, gallium-68 DOTATATE PET-CT, and I-131 MIBG scintigraphy, played a vital role in localization and surgical planning. Laparoscopic excision remains the mainstay of treatment, with favorable postoperative outcomes. Postoperative surveillance with regular biochemical testing and imaging is recommended due to the intermediate metastatic risk associated with the tumor's GAPP score. This case underscores the importance of maintaining a high index of suspicion for paraganglioma in retroperitoneal masses, even in the absence of typical clinical features. 
